# Epigenetic and phenotypic changes result from a continuous pre and post natal dietary exposure to phytoestrogens in an experimental population of mice

**DOI:** 10.1186/1472-6793-8-17

**Published:** 2008-09-15

**Authors:** Carlos M Guerrero-Bosagna, Pablo Sabat, Fernanda S Valdovinos, Luis E Valladares, Susan J Clark

**Affiliations:** 1 Center for Reproductive Biology, School of Molecular Biosciences, Washington State University, Pullman, WA, 99164-4231, USA; 2 Laboratorio de Ecofisiología Animal, Departamento de Ciencias Ecológicas, Facultad de Ciencias, Universidad de Chile, Santiago, Chile; 3 Center for Advanced Studies in Ecology & Biodiversity and Departamento de Ecología, Facultad de Ciencias Biológicas, Pontificia Universidad Católica, de Chile, Santiago, Chile; 4 Laboratorio de Hormonas y Receptores, Instituto de Nutrición y Tecnología de los Alimentos (INTA), Universidad de Chile, Santiago, Chile; 5 Epigenetics Laboratory, Cancer Program, Garvan Institute of Medical Research, Sydney, Australia

## Abstract

**Background:**

Developmental effects of exposure to endocrine disruptors can influence adult characters in mammals, but could also have evolutionary consequences. The aim of this study was to simulate an environmental exposure of an experimental population of mice to high amounts of nutritional phytoestrogens and to evaluate parameters of relevance for evolutionary change in the offspring. The effect of a continuous pre- and post-natal exposure to high levels of dietary isoflavones was evaluated on sexual maturity, morphometric parameters and DNA methylation status in mice. Adult mice male/female couples were fed *ad libitum *either with control diet (standard laboratory chow) or ISF diet (control diet plus a soy isoflavone extract at 2% (w/w) that contained the phytoestrogens genistein and daidzein). In the offspring we measured: i) the onset of vaginal opening (sexual maturation) in females, ii) weight and size in all pups at 7, 14, 21 and 42 days post-natal (dpn) and iii) DNA methylation patterns in skeletal α-actin (*Acta1*), estrogen receptor-α and *c-fos *in adults (42 dpn).

**Results:**

Vaginal opening was advanced in female pups in the ISF group, from 31.6 ± 0.75 dpn to 25.7 ± 0.48. No differences in size or weight at ages 7, 14 or 21 dpn were detected between experimental groups. Nevertheless, at age 42 dpn reduced size and weight were observed in ISF pups, in addition to suppression of normal gender differences in weight seen in the control group (males heavier that females). Also, natural differences seen in DNA methylation at *Acta1 *promoter in the offspring originated in the control group were suppressed in the ISF group. *Acta1 *is known to be developmentally regulated and related to morphomotric features.

**Conclusion:**

This study demonstrates in mammals that individuals from a population subjected to a high consumption of isoflavones can show alterations in characters that may be of importance from an evolutionary perspective, such as epigenetic and morphometric characters or sexual maturation, a life history character.

## Background

The evolutionary origin of new characters in a lineage is considered to be a process different from that of maintenance of these characters through generations [1,2]. Embryonic development plays an important role in the origin of viable phenotypic variation, on which natural selection may act further, thus, from an evolutionary perspective it is important to understand morphogenic processes taking place during early development [3]. Morphological transformations throughout evolutionary history have been produced from context-dependent changes in genetic processes that occur during development [4]. Considering the genome as responsive to environment has led to the hypothesis from us and others that development and/or epigenetics can provide sources of variation that are dependent on the environmental context [5-7]. Moreover epigenetic status in adulthood is directionally dependent on the animal's nutritional status during early development [8,9]. Nevertheless, few studies until recently have devoted attention to environmental compounds that could directly influence early changes implicated in the origin of new characters. Endocrine disruptors (ED) are among those compounds, since they are capable of driving or inducing the occurrence of new characters and/or phenotypes during early development. Endocrine disrupting environmental chemicals may function as estrogens, antiestrogens and antiandrogens, producing reproductive and developmental effects in a variety of organisms [10]. Furthermore, exposure to environmental xenobiotics during early development may have consequences on adult stages [10,11]. In this study we evaluated the effect of dietary exposure to high levels of phytoestrogens on sexual maturity, morphometric parameters and DNA methylation status in mice offspring to determine the potential evolutionary consequences of exposure to a phytoestrogenic based diet in an experimental population of mice.

During embryogenesis, the fetal microenvironment is susceptible to maternal influences due to dietary compounds [12] or changes in maternal hormonal state [13]. Indeed, the maternal hormonal state can be influenced by dietary consumption of natural compounds with estrogenic effects found in vegetables, such as phytoestrogens [14]. Daidzein and genistein are phytoestrogens naturally available (sometimes in high quantities) in food items commonly present in mammals' natural diets such as fruits, nuts or seeds [15], but specially wheat, oats and soy [15,16]. In humans, isoflavone consumption in Asian countries (25 to 100 mg/day) is much higher than in western countries, such as UK, for example, with daily consumption below 1 mg [17]. Mouse commercial diets may contain levels of 21 mg of genistein plus 14 mg of daidzein per 100 g of diet [18], which corresponds to consumption of approximately 1.4 mg of isoflavones/day.

Acting early during development, exogenous estrogenic compounds may also play a role in producing modifications during key stages of ontogeny in mammals [5], having consequences on population traits such as mate preference [19] or life-history traits such as sexual maturation [20]. For example, in mice, prenatal treatment with the synthetic estrogen bisphenol-A (BPA) affects timing of sexual maturation as the number of days between vaginal opening and first vaginal estrous is significantly reduced [20]. Morphological alterations can also be produced in adults due to exposure to estrogenic compounds early during ontogeny. Takai *et al*. [21] have shown that blastocysts exposed to the synthetic estrogen BPA produce adult mice that are heavier at weaning than controls, despite having similar weight at birth. Natural estrogenic compounds, such as genistein, have also been studied, and produce the opposite effect on body mass. For example, rodents fed the phytoestrogen genistein between 1 and 5 days post-natal gave birth to pups with lower post-puberty body weights [14]. In humans, studies on early effects of phytoestrogen consumption are scarce. However, phytoestrogen consumption may delay breast development [22] and would have a protective effect against breast cancer [23]. Nevertheless, the effect may be protective only if the exposure is during childhood/adolescence and would occur through upregulation of breast cancer tumor suppressors such as *BRCA1 *[24]. It is interesting to highlight that transmission of isoflavones from mother to child has been reported in humans [25].

There are many ways by which EDs can regulate gene expression to produce phenotypic changes, [26-28], one of which is epigenetic regulation [29]. For example, EDs have the ability to induce alterations in DNA methylation patterns on key genes and produce related transcriptional changes [30,31]. EDs are capable of triggering changes in DNA methylation during the development of organs. For example, Li *et al *[32] demonstrated that neonatal exposure to DES (diethylstilbestrol) produced abnormalities in the demethylation of the lactoferrin promoter. Moreover, DES administration to pregnant rats during early post-implantation development have been shown to produce a greater susceptibility for specific tumor formation in the rete testis and reproductive tract tissues in F1, which reappears in the non-exposed F2, suggesting epigenetic alterations due to maternal exposure to EDs [33]. Recently, Dolinoy *et al*. [34] have shown that maternal BPA treatment decreases the offspring's CpG DNA methylation in an intracisternal A particle retrotransposon upstream of the *Agouti *promoter, resulting in a change in the phenotype in the coat color pattern. Interestingly, dietary supplementation of the BPA treatment with methyl donors or genistein inhibits the hypomethylating effect of BPA, producing the same coat color pattern observed in the controls [34]. Imprinted genes have also been shown to be target of EDs action. *In vitro *exposure of preimplantation embryos to the contaminant 2,3,7,8-tertra-chlorodibenzo-*p*-dioxin can alter DNA methylation in the *H19 *and *IGF-2 *imprinted genes [35]. Other examples of potential epigenetic modifications due to the action of EDs are those occurring in germ line cells during differentiation. Exposure of rat mothers to either vinclozolin or methoxychlor during embryonic days 8 to 15 produces transgenerational defects in the spermatogenic capacity, which are shown to be transmitted throughout four generations (F1 to F4) and also alteration in methylation patterns in the F1 [36]. Exposure not only to synthetic estrogens but to phytoestrogens has also been shown to influence epigenetic change. For example administration of coumestrol and equol to newborn mice can enhance methylation and produce inactivation of the proto-oncogene *H-ras *[37], and DNA methylation patterns is altered in 8-week-old mice that consumed high doses of genistein [38]. More recently, Dolinoy *et al*. [39] showed that maternal dietary genistein supplementation of mice during gestation shifted the coat color of heterozygous yellow agouti offspring toward pseudoagouti, which is associated with changes in methylation patterns in that gene.

The aim of our study was to simulate an environmental condition of exposure to high amounts of nutritional phytoestrogens on an experimental population of mice and to evaluate potentially important characters for evolutionary change as consequence of such exposure. We investigated population and epigenetic effects on offspring after exposure to an environmental estrogen through treating mice with a continuous pre- and post-natal diet high in phytoestrogens. Life history characters were assessed in the offspring, such as female sexual maturation (measured as the onset of vaginal opening) and offspring sex ratio. Morphometric characters such as weight and size were also measured in all pups. CpG methylation profiles were measured in the promoter region of the skeletal α-actin (*Acta1*), estrogen receptor-α (*ERα*) and *c-fos*, in pancreas and liver of the offspring born from both treatment groups. Liver is an important non classical target for estrogens, in addition to the classic estrogenic response of reproductive organs [40], and is implicated in non diabetic endocrine disorders [41]. In pancreas, in turn, methylation pattern alterations in proto-oncogenes have been reported due to treatment of newborn mice pups with phytoestrogens [37]. Genistein, through an estrogen receptor mediated action, has been reported to modulate gene expression in a variety of tissues in male mice throughout development [42]. *ERα *has been reported to be responsive to *in utero *signalling in mice, since maternal arsenic exposure correlates with increased *ERα *expression and reduced *ERα *promoter DNA methylation in the adult offspring [43]. *Acta1 *is developmentally regulated and associated with morphometric features in mouse [44] and the promoters associated with both *Acta1 *and *ERα *show tissue specific DNA methylation heterogeneity [45,46]. *c-fos*, in turn, is a protooncogene and know to have an estrogen response element that binds the estrogen receptor [47,48] and that may be a key factor in relating estrogenic stimuli to methylation changes [5]. Therefore, we have selected these three different gene promoter regions as surrogate markers to measure if DNA methylation patterns could be altered, during development, by high levels of isoflavones in the maternal diet before and during gestation.

The present paper shows that exposure of an experimental mouse population to nutritional phytoestrogens in the form of an isoflavone extract (not purified phytoetrogens) can advance vaginal opening in female pups, reduce size and weight in adult offspring and suppress normal gender differences in weight seen in controls (males heavier that females). Also, natural gender differences seen in DNA methylation at the *Acta1 *promoter in the offspring born in the control group were suppressed in the group consuming a diet high in isoflavones (ISF diet). This study demonstrates that individuals from a mammalian population subjected to a high consumption of isoflavones can show altered characters, which are relevant from a evolutionary perspective, such as epigenetic and morphometric characters or sexual maturation as a life history character.

## Methods

### Experimental treatments

Adult mice (*Mus musculus*) C3H strain from a lab stock population were initially raised in individual plastic cages on a standard laboratory chow diet for rodents (Champion^® ^S.A., Santiago, Chile) and water *ad libitum *(control diet). Mice were randomly assigned in male/female couples to one of following experimental treatments: mice were fed with i) control diet or ii) control diet plus a commercial concentrate of soy isoflavones (ISF) (Soy Life^®^, Netherlands B. V.) added at 2% (w/w), the ISF diet. To ensure high levels of plasmatic isoflavones in mice fed on the ISF diet, treatment was initiated two weeks before placing the male and female couples in the same cage. The proportion of soy isoflavone concentrate in the diet was chosen considering a previous study [49] in which post weaning long-term consumption of meals with as high as 2.4% soy extract produced significant agonistic effects in a variety of estrogen-dependent tissues and reproductive parameters in female rats, in addition to advancing the age of vaginal opening. Isoflavone concentration in Control and ISF diets (showed in Table 1) was determined by chromatographic analysis as previously described [50]. In both experimental treatments, animals were fed *ad libitum *and maintained at a light cycle of 12:12 at 22 ± 2°C. To avoid maternal cannibalism, pups were not handled until the age of 7 days post-natal (dpn) when gender identification was performed in each litter.

**Table 1:** Isoflavone composition of the experimental diets used in this study

	**Daidzein**	**Genistein**	**Total isoflavones**
**Control diet**	32.06 mg/100 g	15.4 mg/100 g	47.46 mg/100 g
**ISF diet**	160.89 mg/100 g	38.92 mg/100 g	199.81 mg/100 g

### Population characters evaluation

The time of the onset of vaginal opening, expressed as dpn, was used to measure sexual maturation in females. All female pups were examined daily after the age of 20 dpn to check occurrence of vaginal opening. Morphometric parameters such as weight, size and ano-genital distance were measured in all pups at 7, 14, 21 and 42 dpn. The Student's *t *test or Two-way ANOVA was used as statistical test. Two-way ANOVA were used in those comparisons in which diet and gender were tested as independent variables. Statistical analyses were performed with Statistica 6.0 (Statsoft^®^). All protocols used in the present study were approved by the Institutional Animal Care and Use Committee at INTA (Instituto de Nutrición y Tecnología de los Alimentos), Universidad de Chile.

### Animal euthanasia

The offspring used to obtain DNA from pancreas and liver were euthanized after the age of 42 days. All sacrifices were performed according to procedures recommended by the 2000 Report of the American Veterinary Medicine Association (AVMA) Panel on Euthanasia [51]. Animals were placed in a glass chamber with CO_2 _and maintained until 1 minute after no vital signs were observed. Cervical dislocation was then performed to ensure the animal was dead.

### DNA isolation and bisulphite treatment

DNA was extracted with Wizard^® ^DNA Extraction Kit (Promega) from liver and pancreas of adult mice born to females previously assigned to one of the experimental treatments. Bisulphite treatment of DNA was carried out as previously described by Clark *et al*. [52] and Clark and Frommer [53], with modifications described in Warnecke *et al*. [54] and Clark *et al*. [55]. The process of bisulphite treatment exploits the different sensitivities of cytosine and 5-methylcytosine to deamination by bisulphite under acidic conditions, in which cytosine undergoes conversion to uracil, whereas 5-methylcytosine remains unreactive [55]. The bisulphite reaction was desalted using a DNA clean-up column (Promega), as instructed by manufacturer. Bisulphite treated DNA from liver and pancreas was eluted in 50 μl H_2_O.

### PCR conditions

**Figure 1:**
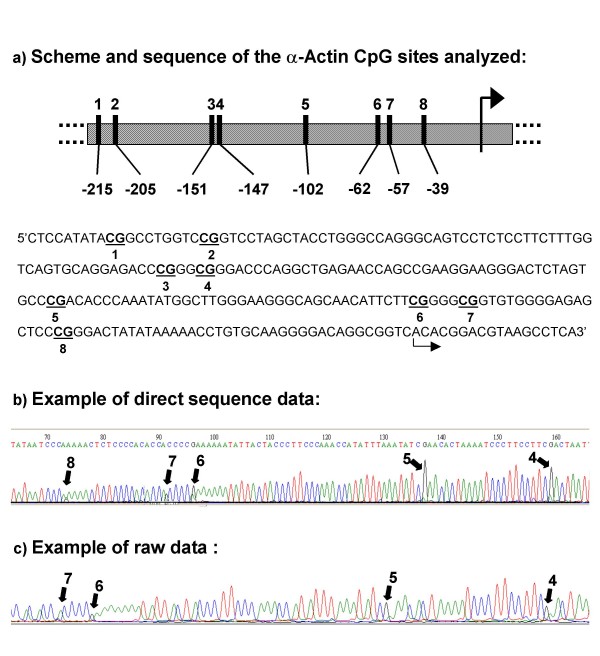
**(a) **scheme shows the distribution of CpG sites in the region analyzed for methylation changes in the promoter of *Acta1 *and their distance to the starting of transcription, describing also the sequence analyzed. **(b) **and **(c) **correspond to sequencing of PCR products amplified from bisulphite treated DNA that includes the 8 CpG sites analyzed, which are indicated with arrows. **(b) **shows electrophenograms with direct sequencing data and **(c) **shows raw data electrophenograms from which CpG DNA methylation was quantified; shown sequences were obtained with the reverse primer; CpG sites in the forward strand appear in the reverse strand as CG when methylated and as CA when unmethylated.

For *Acta1*, the amplified region in the promoter ranged from 529–785, numbers corresponding to GenBank accession no. M12347, as previously reported [45]. CpG site distances to the starting of transcription are shown in (Fig. 1a). Nested PCR primers used were: forward external, 5'-AAGTAGTGATTTTTGGTTTAGTATAGT-3'; reverse external, 5'-ACTCAATAACTTTCTTTACTAAATCTCCAAA-3'; forward internal, 5'-GGGGTAGATAGTTGGGGATATTTTT-3'; reverse internal, 5'-CCTACTACTCTAACTCTACCCTAAATA-3'.

**Figure 2:**
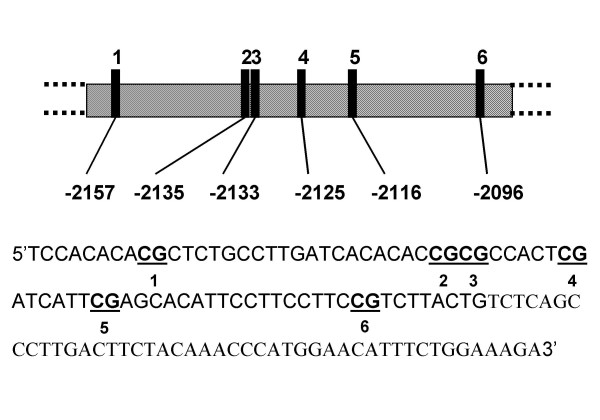
Scheme shows the distribution of CpG sites in the region analyzed for methylation changes in the promoter of *ERα*, the distance to the starting of transcription of the first translated exon and the sequence analyzed; the region includes 6 CpG sites.

For *ERα*, the amplified fragment in the promoter was in the 5' flanking region (in the untranslated exon C) [56] and ranged from 282–608, numbers corresponding to GeneBank accession no. AJ276597. CpG site distances to the start of the first translated exon are shown in (Fig. 2). The following semi-nested PCR primers were used: forward external, 5'-GAGTTTTTTTTAGGAATGTTGATTTT-3'; forward internal, 5'-GGAGGGGTTGTTAAGTGTTTT-3'; reverse, 5'-ACACAACTTCCTTCTCCAACTAAAAA-3'.

For *c-fos*, the amplified fragment in the promoter region ranged from 226–669, numbers corresponding to GeneBank accession no. V00727. The following semi-nested PCR primers were used: forward, 5'-AGGGGTAGATATTGGTGGGAGTTGT-3'; reverse external, 5'-CTACACCCTCAAAATTAACTACAACC-3'; reverse internal, 5'-CCTCCTTTACACAAAATATCCATATTAAA-3'.

PCR reactions were performed in a final volume of 20 μl, containing 10 μl 2× Promega master mix, 7 μl dnase free water, 1 μl of each reverse and forward primers and 1 μl of bisulphite treated DNA template (~40 ng of DNA). PCR was programmed as follows: 94°C/2 min × 1 cycle; 94°C/1 min, annealing temperatute/1 min, 72°C/3 min, × 5 cycles; 94°C/0.5 min, annealing temperatute/2 min, 72°C/1.5 min, × 25 cycles; 72°C/2 min × 1 cycle. Annealing temperatures were, 55°C for both rounds of amplification for *Acta1*; 63°C for the first round primers (forward external and reverse) and 59°C for the second round primers (forward internal and reverse) for *ERα*; 60.5°C for the first round primers (forward and reverse external) and 59°C for the second round primers (forward and reverse internal) for *c-fos*. All PCR reactions were performed in triplicate for each tissue sample.

### DNA sequencing

PCR products were purified by the Exo-Sap method (Exonuclease I and Shrimp Alkaline Phosphatase from Amersham Biosciences). The sequencing reactions were performed by using the Dye Terminator cycle sequencing kit with AmpliTaq DNA polymerase FS (Applied Biosystems) and the automated 373A NA Sequencer (Applied Biosystems). DNA methylation profiles were analyzed by direct semi-quantitative bisulphite PCR sequencing [55]. The height of the cytosine peak relative to the thymine peak was used to calculate the percentage of methylation for each CpG site as previously described [55,57]. Analysis of the direct PCR sequencing data was performed on raw data electrophenograms (Figures 1b and 1c) in order to improve the sensitivity of the methylation detection. Measurements of raw data peak heights were performed either manually or using the BioEdit Sequence Alignment Editor (Isis Pharmaceuticals, Inc) [58], which permits a fast and accurate measurement of the peak heights. This approach has recently been used to address methylation differences in a recent study [59]. We also cloned and sequenced some of the PCR amplicons to confirm the methylation patterns observed with direct sequencing measured through raw data [see Additional file 1]. We used multivariate ANOVA (MANOVA) to test for changes in methylation. The multivariate approach tests for general changes; when this is significant, univariate approach tests for CpG site punctual effects. Statistical analyses were performed with Statistica 6.0 (Statsoft^®^).

## Results

### Effect of a diet of soy isoflavones on sexual maturation and morphometric parameters

**Figure 3:**
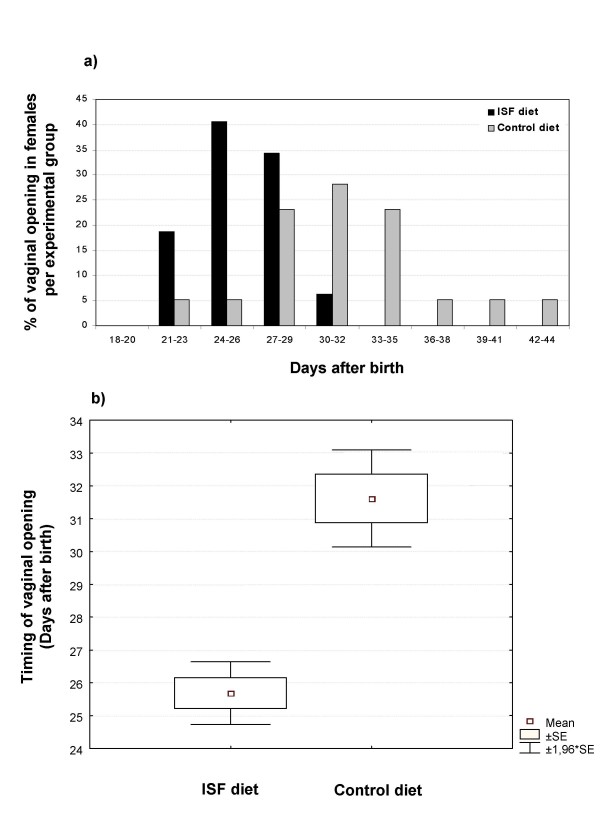
Timing of female mice sexual maturation observed in the offspring of a population of mice subjected to ISF or control diet, expressed as (**a**) distribution of occurrence of vaginal opening along days after birth and as (**b**) mean day of occurrence of vaginal opening after birth ± SE ± 1,96*SE (P < 0.001).

The content of the isoflavones genistein and daidzein were measured in both experimental diets supplied to mice and is shown in Table 1. Mice fed a continuous diet of isoflavones (ISF diet) displayed no difference to the control groups in sex ratio or litter size. Average sex ratio of males found in litters born to parents treated with the ISF diet was 49.4 % ± 5.8 (n = 12) and in litters born to parents fed on control diet was 53.2 % ± 4.6 (n = 13) (values expressed as means of % of males per litter ± SE; *P *= 0.63). Litter size values were 8.1 ± 0.69 (n = 12) for the ISF group and 8.5 ± 0.58 (n = 13) for the control group (values expressed as means of litter size ± SE; *P *= 0.68). However, sexual maturation was advanced by approximately 6 days in female pups born to ISF-fed mothers, with vaginal opening occurring at 25.7 dpn ± 0.48 (n = 32) as compared to 31.6 dpn ± 0.75 (n = 39) in the control group (values expressed as mean day of vaginal opening ± SE; *P *< 0.001) (Figures 3a and 3b). Not surprisingly, weight, size and ano-genital distance in females at the day of vaginal opening were also reduced in the ISF feeding group. Weight changed from 17.9 g ± 0.11 (n = 39) to 12.6 g ± 0.09 (n = 32), size from 8.1 cm ± 0.08 (n = 39) to 7.2 cm ± 0.14 (n = 32), and ano-genital distance from 6.6 mm ± 0.45 (n = 39) to 5.8 mm ± 0.54 (n = 27) (all values are expressed as mean ± SE; *P *< 0.001 for all comparisons). Such decreases are presumably associated with the reduced age at which females mature.

**Figure 4:**
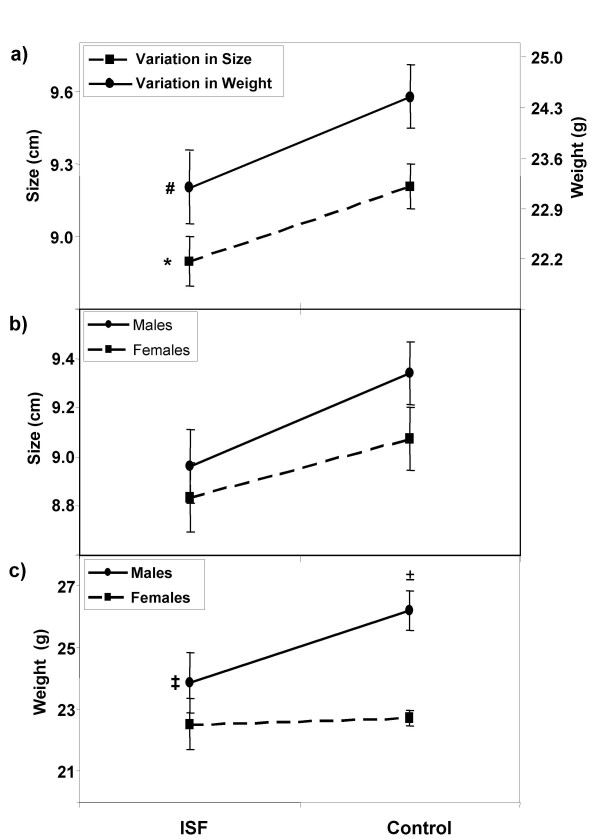
**Comparison of morphometric parameters between pups 42 days old.** Variation in adult weight and size observed in the offspring of a population of mice subjected to ISF or control diet is shown. (**a**) indicates the general trend of decreased size (*P *= 0.03, *) and weight (*P *= 0.06, **#**) in the ISF group with regard to controls, when pooling males and females. Size differences in (**a**) are explained by variations in both sexes, as shown in (**b**). Weight differences in (**a**) are accounted for by variations only in males, which are heavier in the control group than in the ISF group (*P *= 0.04, **‡**), as can be seen in (**c**). Also, in the control group males are heavier that females (*P *< 0.001; **c**, right, ±), difference that is suppressed in the ISF group (*P *= 0.3; **c**, left).

When pooling males and females, no differences were detected in the offspring among experimental groups in size or weight at ages 7, 14 or 21 dpn. However, reduced size (*P *= 0.03) and weight (*P *= 0.06) (2-way ANOVA; n_ISF _= 19, n_CONTROL _= 24) were observed at age 42 dpn in the ISF group (Fig. 4a). Contribution of both sexes variation account for size differences observed, as shown in Figure 4b, because no significant changes were detected independently for each gender (males, *P *= 0.09, Students *t *test; n_ISF _= 9, n_CONTROL _= 12; females, *P *= 0.19, Students *t *test; n_ISF _= 10, n_CONTROL _= 12). Weight differences, in turn, are accounted for by variations only in males (*P *= 0.04, Students *t *test; n_ISF _= 9, n_CONTROL _= 12), but not in females (*P *= 0.8, Students *t *test; n_ISF _= 10, n_CONTROL _= 12), as seen in Figure 4c. Thus, normal gender differences in weight seen in the control group (males heavier that females, *P *< 0.001; Student's *t *test; n_FEMALES _= 12, n_MALES _= 12; Fig. 4c, right) are suppressed in the ISF group (*P *= 0.3; Student's *t *test; n_FEMALES _= 10, n_MALES _= 9; Fig. 4c, left). Finally, no differences were detected among experimental groups in ano-genital distance for either males or females.

### Effect of dietary soy isoflavones on DNA methylation

We determined the methylation profile of 8 CpG sites for *Acta1 *(Fig. 1a), 6 CpG sites for *ERα *(Fig. 2) and 18 CpG sites for *c-fos *by using direct bisulphite DNA sequencing across their promoter regions, in both liver and pancreas from male and female pups born to mice fed on ISF and control diets. We previously have shown that this region of *Acta1 *exhibits DNA methylation heterogeneity [45] and therefore is a good candidate to evaluate if the parental diet can result in a tissue or gender specific effect on DNA methylation of the offspring. *ERα*, in turn, has been reported to be responsive to maternal exposure in terms of changes in methylation [43]. *c-fos *is a protooncogene that has an estrogen response element that binds the estrogen receptor [47,48] and may be important in relating estrogenic stimuli to methylation changes [5].

**Figure 5:**
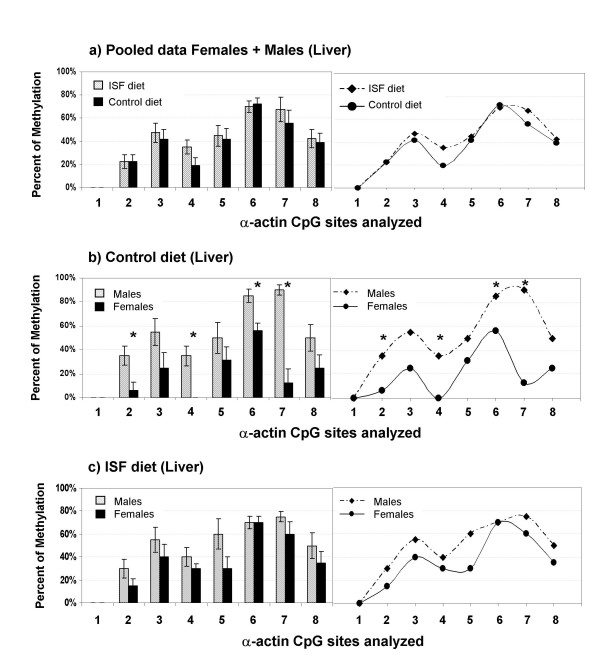
**Methylation profiles of the analyzed region of the *Acta1 *promoter in liver from offspring generated in the control or ISF group.** No differences in DNA methylation in liver were detected among treatments when comparisons were performed pooling male and female data (*P *= 0.3), as shown in **(a)**. Nevertheless, gender methylation differences seen in **(b)**, the controls (*P *= 0.0006), are suppressed in **(c)**, the ISF group (*P *= 0.4). Significant changes in individual CpG sites are shown with *, *P *values are indicated in the text.

For *Acta1*, MANOVA showed no differences in DNA methylation in liver between pups born to mothers fed with the ISF or the control diet when comparisons were performed within males (Wilks Lambda: F = 0.1, *P *= 0.6; n_ISF _= 5, n_CONTROL _= 5), within females (Wilks Lambda: F = 10.2, *P *= 0.2; n_ISF _= 5, n_CONTROL _= 4; Fig. 5a) or pooling male and female data (Wilks Lambda: F = 1.37, *P *= 0.3; n_ISF _= 10, n_CONTROL _= 9). Nevertheless, overall and individual CpG site specific differences in *Acta1 *DNA methylation that were seen between males and females in the control diet fed mice (Wilks Lambda: *P *= 0.055; Univariate analysis: site 2, F = 10.55, *P *= 0.015; site 4, F = 25.41, *P *= 0.0015; site 6, *P *= 0.015, F = 10.55; site 7, F = 35.6, *P *= 0.0006; n_FEMALES _= 4, n_MALES _= 5; Fig. 5b) were suppressed in the ISF group (Wilks Lambda: F = 1.81, *P *= 0.4; n_FEMALES _= 5, n_MALES _= 5; Fig. 5c). This could be explained by a subtle decrease in the level of methylation in ISF males together with a subtle increase in the level of methylation in ISF females.

**Figure 6:**
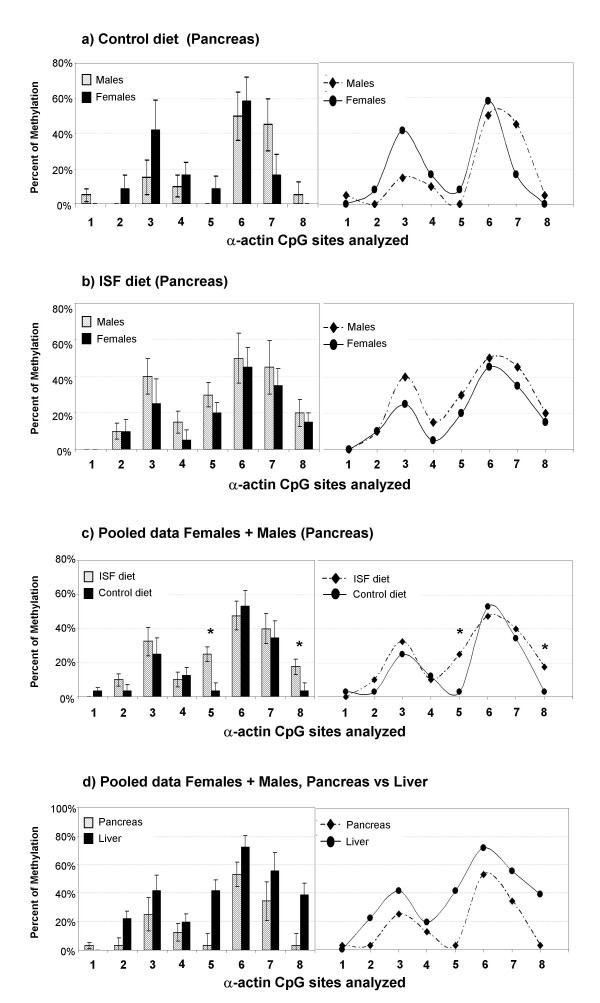
**Methylation profiles of the analyzed region of the *Acta1 *promoter in pancreas from offspring generated in the control or ISF group.** DNA methylation gender differences are not observed in the population maintained on (**a**), control diet (*P *= 0.93), or on (**b**) ISF diet (*P *= 0.837). Nevertheless, pooling male and female data together shows a nearly significant difference due to the ISF treatment (*P *= 0.08), in which CpG site specific changes were detected in sites 5 (*P *= 0.004) and 8 (*P *= 0.045), as seen in **(c), **indicated with *. Using only control animals (both males and females) we found that methylation is increased in liver regarding to pancreas (*P *= 0.007), which shows the occurrence of tissue specific differences in DNA methylation for *Acta1*, as seen in **(d)**.

For *Acta1 *in pancreas, MANOVA revealed no gender differences in the control group (Wilks Lambda: F = 0.2, P = 0.93, Fig. 6a; n_FEMALES _= 3, n_MALES _= 5) or in the ISF group (Wilks Lambda: F = 0.42, P = 0.837, Fig. 6b; n_FEMALES _= 5, n_MALES _= 5). Also, no differences were observed between experimental groups within males (Wilks Lambda: F = 0.5, P = 0.804) or females (Wilks Lambda: P = 0.196, F = 14.83). Pooling male and female data together (Fig. 6c), however, revealed a nearly significant difference with the multivariate approach (Wilks Lambda: F = 2.698, *P *= 0.08; n_ISF _= 10, n_CONTROL _= 8), suggesting that overall changes in methylation are taking place in *Acta1 *from pancreas. This is reinforced by the fact that two CpG site specific changes in methylation were detected, in sites CpG5 (F = 11.168, *P *= 0.004) and CpG8 (F = 4.726, *P *= 0.045), showing increased levels of methylation in the ISF group with respect to the control group. Additionally, we compared the level of methylation between pancreas and liver, using only control animals (both males and females), in order to address the occurrence of tissue specific DNA methylation for *Acta1*. We found that methylation is increased in liver regarding to pancreas (Wilks Lambda: F = 3.509, *P *= 0.047; Univariate analysis: CpG2, F = 6.431, P = 0.022; CpG5, F = 11.727, *P *= 0.004; CpG8, F = 9.926, *P *= 0.007; Fig. 6d; n = 8).

**Figure 7:**
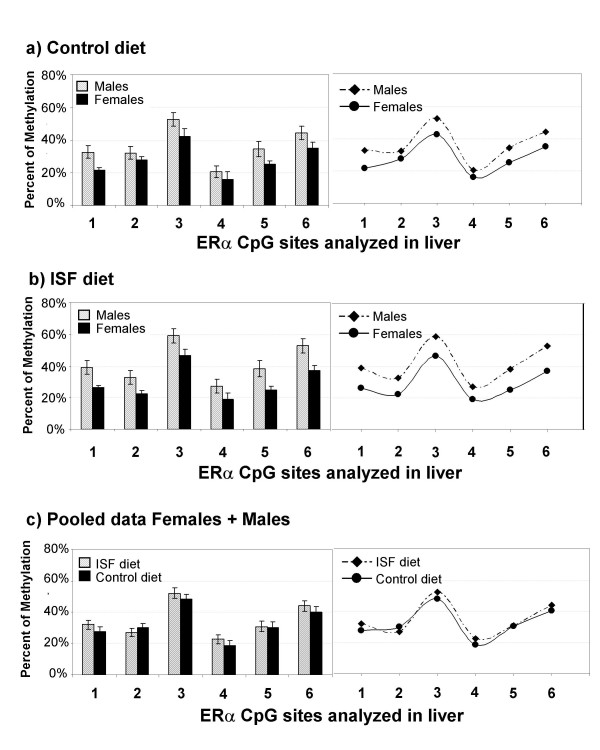
**Methylation profiles of the analyzed region of the *ERα *promoter in liver from offspring generated in the control or ISF group.** No treatment effects were detected within males, within females or pooling male and female data, as seen in (**a**), (**b**) and (**c**).

For *ERα *promoter, the methylation profile of 6 CpG sites located from -2285 to -1979 was analyzed (Fig. 2). In liver, MANOVA showed no gender differences in the ISF group (Wilks Lambda: F = 1.86, *P *= 0.326; n_FEMALES _= 5, n_MALES _= 4) or in the control (Wilks Lambda: F = 8.98, *P *= 0.1; n_FEMALES _= 4, n_MALES _= 5). Also, no multivariate effects of the treatment were detected within males (F = 2.054, *P *= 0.49; n_ISF _= 4, n_CONTROLS _= 5), within females (F = 1.49, P = 0.56; n_ISF _= 5, n_CONTROLS _= 4) or pooling male and female data (F = 2.054, *P *= 0.49; n_ISF _= 9, n_CONTROLS _= 9) (Figures 7a–c).

In addition, we found tissue specific DNA methylation differences for *ERα*, where CpG sites were differentially methylated in liver (Figures 7a–c), but completely unmethylated in pancreas in both experimental groups (n = 4). A lack of methylation across the promoter of *c-fos *was observed in the liver and pancreas for both the ISF and control animals (n = 4) (data not shown).

Our data has demonstrated that a diet rich in phyoestrogen can result in an advancement of sexual maturation in female pups as well suppress normal gender differences in the DNA methylation pattern of a tissue specific methylated gene such as *Acta1*. These results support the hypothesis that alterations in the hormonal state of the pregnant females produced by a diet of phytoestrogens or other xenoestrogens can affect phenotype as well as the epigenetic state of the offspring.

## Discussion

The present study evaluated aspects related to morphology, life-history traits and epigenetic modifications in DNA methylation in the offspring of an experimental population of mice subjected to a high dietary consumption of isoflavones. We previously hypothesized that treatment with dietary isoflavones could alter the hormonal microenvironment where the fetus develops, which could, in turn, trigger changes in early development, leading to phenotypic and population changes, which under certain conditions may have evolutionary relevance [5]. If a given environmental compound is persistently present generation after generation in a population of individuals, it may lead to a consistent altering of parameters and characters in that population [29].

In this study we first evaluated sex ratio, a life-history trait, in offspring exposed to the ISF diet, since previous evidence has demonstrated that early hormonal exposure to different levels of testosterone can induce sex ratio alterations in litters [60]. In contrast, we did not find significant differences in the percent of males in litters between the control and ISF groups, suggesting that eventual effects of phytoestrogens do not interfere with the mechanisms of sex determination. Another life-history character analyzed was female sexual maturation. We found that maternal exposure to dietary isoflavones advanced puberty in female offspring. This result confirms and complements what other studies have found in mice. For example, a similar trend was observed with prenatal treatment with BPA, which is shown to reduce the days between the onset of vaginal opening and the first vaginal estrous [20]. Another study that included phytoestrogens in the diet between 15–30 days post partum, showed advanced vaginal opening [61]. In rats, the age at vaginal opening was also reported to be reduced due to dietary consumption of isoflavones after weaning [49]. Although further studies should be performed in order to clarify when advancement in sexual maturation is triggered (before or after birth, or before or after weaning), these data suggest that perinatal phytoestrogen dietary consumption is an important factor in advancing sexual maturation in females rodents.

**Figure 8:**
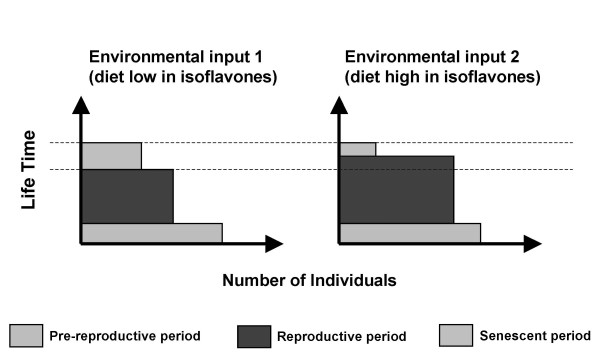
Scheme showing two hypothetical population structures subjected to different consumption of dietary isoflavones.

Steroid-mediated maternal effects in viviparous organisms may also have long-lasting consequences on life-history aspects of the offspring and be an important evolutionary factor [62]. Sexual maturation is an important life-history character in animal populations, and when changed, may lead to altered population structure composition. Compared to other life history traits, changes in the timing of sexual maturation strongly impact on fitness [63]. For example, organisms in which sexual maturation occurs earlier have a high probability of surviving to maturity. Advanced age of maturity will also produce shorter juvenile periods [63] and shorter generations [63,64]. Although timing of maturation is thought to evolve as a consequence of selective pressures [63], which implies restrictive environmental forces acting on organisms, here we show that the environment, rather than restricting the ontogeny of organisms, can act inducing changes in life-history traits, which may also lead to population and evolutionary changes. Figure 8 illustrates how the structure of a population, expressed in terms of reproductive periods, may be affected by an environmental stimulus such as dietary isoflavones. Moreover, if both the stimulus and the induced population changes are conserved throughout generations, an evolutionary change could be expected [5].

Regarding morphometric aspects, we found that male pups born within the ISF group have reduced weight and size in comparison to the control group, at the age of 42 dpn. This is concordant with results found by Nagao *et al*. [14] that show lower body weight in mice pups born to mothers injected with the phytoestrogen genistein. Our results however, show the opposite effect of that reported by Takai *et al*. [21], in which culturing blastocytes in presence of the synthetic estrogen BPA resulted in heavier pups at weaning, as compared to controls. This could be due to the source of the estrogen (natural or synthetic) employed in these studies. It is likely that environmental estrogens, such as genistein, would produce effects other than those produced with synthetic estrogens, such as BPA. The mechanism behind the weight and size differences could either be due to epigenetic or physiological changes. The sex specific effect in weight shown here may be related to testosterone levels, since a strong correlation between body mass and testosterone levels is known to exist. For example, serum testosterone is about 9.6-fold higher in mice selected for high body weight [65]. Interestingly, phytoestrogens are shown to reduce the conversion from androgens to estrogens through inhibiting aromatase activity in human granulose-luteal cell cultures [66,67], which will produce imbalances in the testosterone/estradiol ratio. Further investigation should be done in order to elucidate if this male related weight response to phytoestrogens is dependent on testosterone production.

Taking into consideration the hypothesis that there is an epigenetic basis for the differences in weight as a result of ISF treatment, we could expect that these gender-specific differences in weight and size could be related to gender-specific changes in DNA methylation. We analyzed the promoter region of *Acta1 *as a surrogate marker for DNA methylation change, as we previously reported that regulation of this gene is associated with tissue specific DNA methylation [45]. In this new study we found that the consumption of high amounts of dietary phytoestrogens by mice mothers can lead to sex-specific changes in DNA methylation patterns of the *Acta1 *promoter, specifically in the liver. Interestingly, we discovered that there is a natural difference between males and females in *Acta1 *promoter DNA methylation, as males showed higher levels of methylation than females. Treatment with dietary isoflavones appears to suppress such gender differences, decreasing the level of DNA methylation in males and/or increasing it in females. These findings pose an intriguing question in terms of what is the mechanism behind the gender differences in DNA methylation. In addition to the differences observed in liver, we also found that DNA methylation changes occurred in the pancreas. As expected, we found that methylation of *Acta1 *in pancreas shows a different tissue-specific profile than in liver, as previously reported in Warnecke & Clark [45]. However, analysis of male and female pooled DNA samples showed increased methylation in response to ISF treatment in two CpG sites in pancreas, CpG5 and CpG8, suggesting that there is a potential sequence-specific and tissue-specific effect as a result of diet. Tissue specific differences in methylation profiles were also detected for *ERα*, which show absence of methylation in pancreas, contrasting with a well-defined methylation profile seen in liver. The phenotypic consequences of changes in methylation in the *Acta1 *promoter could be related to the changes in male weight. Crawford et al. [44] showed that null *Acta1 *mice die in the early neonatal period and, moreover, 4 days after birth they show a markedly lower body weight than normal littermates. Although the phenotypic effect of DNA methylation in males is not expected to be as drastic as that seen in *Acta1 *null mice, we could expect that altering DNA methylation in the *Acta1 *promoter in some organs in males could contribute to body weight reduction.

**Figure 9:**
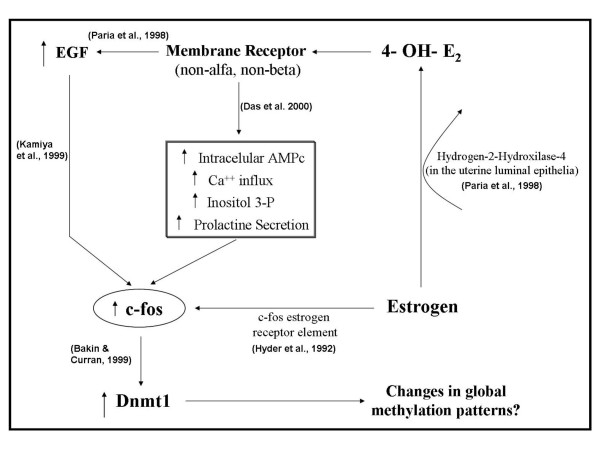
Scheme of a proposed mechanism of action through which endocrine disruptors could alter methylation patterns acting from the mother to the embryo.

Considering these aspects, the main question is 'what is the mechanism through which the intrauterine environment could influence DNA methylation in developing embryos?' We have previously hypothesized that phytoestrogenic influences could take place either directly, through the presence of isoflavones in uterine secretions, or indirectly, mediated by other compounds secreted in the uterine epithelia such as 4-OH-17β-estradiol, responding to circulating levels of isoflavones [5]. It is not known whether compounds with estrogenic action, like isoflavones, inside the uterus could act directly upon the developing embryo. Nevertheless, it is possible that the relationship between such estrogenic stimuli and methylation in the preimplantational embryo is mediated by the expression of *c-fos*. It has been reported that *c-fos *directly regulates the transcription of the DNA maintenance methyltransferase gene, *Dnmt1*, increasing the enzyme levels [68]. On the other hand, the induction of *c-fos *is attributed to membrane-mediated estrogen actions [69]. Through this mechanism, which provides an alternative pathway to the classical estrogen receptors α and β, estrogenic compounds could trigger responses, as has been observed in pancreatic β cells [70]. Thus, the membrane-mediated estrogenic actions would first induce *c-fos *and then trigger the activation of the *Dnmt1 *enzyme. Furthermore, in blastocysts, this indirect and membrane mediated mechanism of *c-fos *activation could also occur. In preimplantational blastocysts, latent blastocysts can be activated in presence of 4-OH-17β-estradiol, a catecholestrogen synthesized from 17β-estradiol in uterine luminal epithelia by the action of the hydrogen-2/hydroxylase-4 enzyme [71]. This response to 4-OH-17β-estradiol could also occur via a pathway distinct from the classical nuclear estrogen receptors [71]. Levels of 4-OH-17β-estradiol increase with the epithelial growth factor (EGF) receptor [71]. Interestingly, other studies have demonstrated that an increase in the EGF receptor may also be related to activation of *c-fos *[72]. A direct induction of *c-fos *by estrogen has also been shown in different cell types [73,74], which occurs via an estrogen receptor element present in this gene [47]. Thus, estrogenic stimuli could induce *c-fos*, either directly, through a gene receptor, or indirectly through membrane-mediated reactions. Figure 9 summarizes the possible pathways for the estrogenic stimulus to influence DNA methylation in developing embryos.

Taking this background into consideration, we analyzed DNA methylation in the promoter region of *c-fos*, but found that the *c-fos *promoter was unmethylated. Therefore, if *c-fos *expression is regulated by an estrogenic stimulus it is possible that methylation is not involved in its regulation, but this does not exclude the possibility that i) other mechanisms of epigenetic regulation may be occurring, since for example, changes in chromatin structure or histone modifications may also be affected following estrogenic stimuli [29] and that ii) *c-fos *can still be acting as a mediator in the process of methylation of other genes. The involvement of EDs in the process of DNA methylation is also supported by the finding that exposure of early embryos to TCDD, DES, or PCB 153 alters the DNA methyltransferase activity, which has the potential to induce a change in methylation status of genes and to affect further developmental processes [75]. Thus, the link relating EDs (including phytoestrogens) and DNA methylation is gaining increased support. Future work should be performed in order to investigate if other genes are altered in their DNA methylation patterns as a result of isoflavones exposure or other environmental signals capable of acting across the uterine barrier and affecting the developing embryo. Particularly interesting would be the study of the *de novo *and maintenance DNA methyltransferases. However, future focus should take into consideration that changes in methylation may be sex-specific.

## Conclusion

The present study demonstrates, in mammals, that a population subjected to a high consumption of isoflavones can show a variety of alterations in its individuals, including changes in epigenetic and morphometric characters and in sexual maturation. The results obtained here, together with previous reports in the field ([8], [12-14], [21], [23-26], [28-31], [41], [57], [58]), start to reveal important facts about the role of EDs (including natural compounds such as phytoestrogens) on altering epigenetic mechanisms such as DNA methylation. Consumption of compounds such as phytoestrogens by pregnant mothers may interfere with the establishment of DNA methylation in the developing embryos, leading to a change in DNA methylation and potential gene expression patterns in the resulting adults [5]. This is coincident with the idea that heritable variation is sometimes directed and developmentally produced as a response to the environment [6].

As shown here, exposure to environmental estrogens have consequences on characters that are important from the population structure point of view such as sexual maturation. Moreover, ED-induced changes in DNA methylation are interesting from an evolutionary perspective because they could lead to biased mutations as consequences of persistent changes in DNA methylation through generations. It is known, for example, that the methylated form of CpG has a 12-fold higher mutation rate to TpG and CpA [76]. Environmental stimuli could have a great importance in inducing evolutionary change, although some restrictions may apply in order for this process to occur, as we have previously described [5]: i) environmental stimuli could act only on certain key periods in the ontogeny of the organisms in order to produce epigenetic effects; ii) not every but only particular environmental agents acting as such stimuli would be able to produce those epigenetic effects, and iii) a persistent genomic change should be produced generation after generation in a whole population exposed to such stimuli. That persistent change could be achieved by a persistent action of the stimuli, producing a consistent epigenetic change generation after generation, or by altering the genome through mutations or epigenetic changes in imprinted genes, which would express those changes in futures generations.

## Authors' contributions

CG conceived the study, conducted all the experiments and drafted the manuscript. PS participated in the experimental design, analysis of data and drafting the manuscript. FSV collected part of the data related to morphometric characters and sexual maturation. LV participated in the experimental design, analysis of data and drafting of the manuscript. SJC participated in the experimental design, analysis of data and drafting of the manuscript. All authors read and approved the final manuscript.

## Supplementary Material

Additional file 1 Comparison of methylation in *ERα *promoter in liver. Methylation in the same samples was compared with two different procedures after bisulphite conversion: direct sequencing measured by raw data versus cloning and sequencing. This comparison was randomly performed in three samples in order to verify the reproducibility of direct sequencing measured by raw data with regard to cloning and sequencing. Left figures show cloning results and right plots show the comparison with direct sequencing measured by raw data. Methylated CpG sites are indicated as black circles (●) and unmethylated CpG sites as white circles (○). The sample number representing each animal is shown in the figure: **(a) **animal 1 (male, control); **(b) **animal 2 (female, control); and **(c) **animal 23 (female, ISF). Click here for file
