# Supplementary material for: Epigenetic and phenotypic changes result from a continuous pre and post natal dietary exposure to phytoestrogens in an experimental population of mice
Source: BMC Physiol. 2008 Sep 15;8:17. doi: 10.1186/1472-6793-8-17 (PMC2556694; doi:10.1186/1472-6793-8-17)
Supplement: Additional file 1 — Comparison of methylation in ERα promoter in liver. Methylation in the same samples was compared with two different procedures after bisulphite conversion: direct sequencing measured by raw data versus cloning and sequencing. This comparison was randomly performed in three samples in order to verify the reproducibility of direct sequencing measured by raw data with regard to cloning and sequencing. Left figures show cloning results and right plots show the comparison with direct sequencing measured by raw data. Methylated CpG sites are indicated as black circles (●) and unmethylated CpG sites as white circles (○). The sample number representing each animal is shown in the figure: (a) animal 1 (male, control); (b) animal 2 (female, control); and (c) animal 23 (female, ISF). [file 1472-6793-8-17-S1.ppt]

## Slide 1
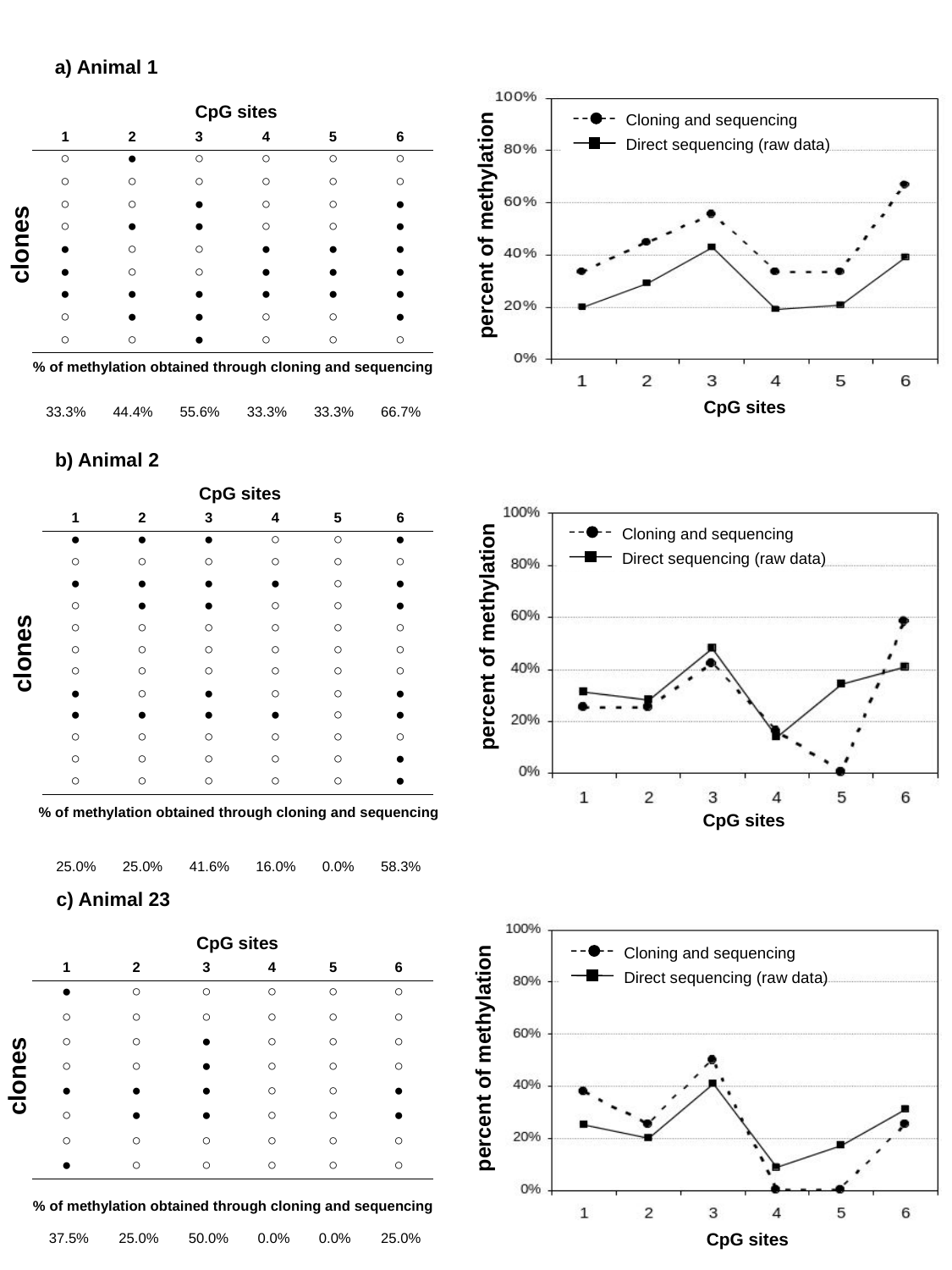

a) Animal 1
Cloning and sequencing
Direct sequencing (raw data)
percent of methylation
CpG sites
CpG sites
| 1 | 2 | 3 | 4 | 5 | 6 |
| --- | --- | --- | --- | --- | --- |
| ○ | ● | ○ | ○ | ○ | ○ |
| ○ | ○ | ○ | ○ | ○ | ○ |
| ○ | ○ | ● | ○ | ○ | ● |
| ○ | ● | ● | ○ | ○ | ● |
| ● | ○ | ○ | ● | ● | ● |
| ● | ○ | ○ | ● | ● | ● |
| ● | ● | ● | ● | ● | ● |
| ○ | ● | ● | ○ | ○ | ● |
| ○ | ○ | ● | ○ | ○ | ○ |
| | | | | | |
| 33.3% | 44.4% | 55.6% | 33.3% | 33.3% | 66.7% |
clones
% of methylation obtained through cloning and sequencing
b) Animal 2
CpG sites
Cloning and sequencing
Direct sequencing (raw data)
percent of methylation
CpG sites
| 1 | 2 | 3 | 4 | 5 | 6 |
| --- | --- | --- | --- | --- | --- |
| ● | ● | ● | ○ | ○ | ● |
| ○ | ○ | ○ | ○ | ○ | ○ |
| ● | ● | ● | ● | ○ | ● |
| ○ | ● | ● | ○ | ○ | ● |
| ○ | ○ | ○ | ○ | ○ | ○ |
| ○ | ○ | ○ | ○ | ○ | ○ |
| ○ | ○ | ○ | ○ | ○ | ○ |
| ● | ○ | ● | ○ | ○ | ● |
| ● | ● | ● | ● | ○ | ● |
| ○ | ○ | ○ | ○ | ○ | ○ |
| ○ | ○ | ○ | ○ | ○ | ● |
| ○ | ○ | ○ | ○ | ○ | ● |
| | | | | | |
| 25.0% | 25.0% | 41.6% | 16.0% | 0.0% | 58.3% |
clones
% of methylation obtained through cloning and sequencing
c) Animal 23
Cloning and sequencing
Direct sequencing (raw data)
percent of methylation
CpG sites
CpG sites
| 1 | 2 | 3 | 4 | 5 | 6 |
| --- | --- | --- | --- | --- | --- |
| ● | ○ | ○ | ○ | ○ | ○ |
| ○ | ○ | ○ | ○ | ○ | ○ |
| ○ | ○ | ● | ○ | ○ | ○ |
| ○ | ○ | ● | ○ | ○ | ○ |
| ● | ● | ● | ○ | ○ | ● |
| ○ | ● | ● | ○ | ○ | ● |
| ○ | ○ | ○ | ○ | ○ | ○ |
| ● | ○ | ○ | ○ | ○ | ○ |
| | | | | | |
| 37.5% | 25.0% | 50.0% | 0.0% | 0.0% | 25.0% |
clones
% of methylation obtained through cloning and sequencing
